# Comparison of the Zebrafish Embryo Toxicity Assay and the General and Behavioral Embryo Toxicity Assay as New Approach Methods for Chemical Screening

**DOI:** 10.3390/toxics8040126

**Published:** 2020-12-21

**Authors:** John C. Achenbach, Cindy Leggiadro, Sandra A. Sperker, Cindy Woodland, Lee D. Ellis

**Affiliations:** 1 Aquatic and Crop Resource Development, National Research Council of Canada, Halifax, NS B3H 3Z1, Canada; jc.achenbach@nrc.ca (J.C.A.); cindy.leggiadro@nrc.ca (C.L.); ssperker5@gmail.com (S.A.S.); 2 New Substances Assessment Control Bureau, Health Canada, Ottawa, ON K1A 0K9, Canada; cindy.woodland@canada.ca

**Keywords:** zebrafish larvae, toxicity assay, phenotype, developmental period

## Abstract

The movement away from mammalian testing of potential toxicants and new chemical entities has primarily led to cell line testing and protein-based assays. However, these assays may not yet be sufficient to properly characterize the toxic potential of a chemical. The zebrafish embryo model is widely recognized as a potential new approach method for chemical testing that may provide a bridge between cell and protein-based assays and mammalian testing. The Zebrafish Embryo Toxicity (ZET) model is increasingly recognized as a valuable toxicity testing platform. The ZET assay focuses on the early stages of embryo development and is considered a more humane model compared to adult zebrafish testing. A complementary model has been developed that exposes larvae to toxicants at a later time point during development where body patterning has already been established. Here we compare the toxicity profiles of 20 compounds for this General and Behavioral Toxicity (GBT) assay to the ZET assay. The results show partially overlapping toxicity profiles along with unique information provided by each assay. It appears from this work that these two assays applied together can strengthen the use of zebrafish embryos/larvae as standard toxicity testing models.

## 1. Introduction

Alternatives to traditional toxicity testing models continue to be sought as more humane end points that accommodate the “3R” principles of replacement, reduction and refinement and are the cornerstone of the laboratory animal science community. While the movement from mammalian models to cell line testing can often fulfil these requirements, it is sometimes difficult to ascertain the physiological relevance of the findings due to the loss of the ability to assess the interplay between different cell types in different tissues. Whole organism models that can bridge the gap between the high-throughput, low-cost, cell-line testing models and the low-throughput, costly, mammalian models are thus still required. The zebrafish (*Danio rerio*) is an established alternative model for toxicity testing that can provide a medium-throughput, cost-effective model that can also provide more information than can be obtained from cell line testing alone [1]. Additionally, it has been established that until zebrafish reach the protected animal stage at 5 days post fertilization (dpf) they can be considered an alternative model to animal testing [2,3,4,5,6,7,8,9]. The zebrafish larval model of toxicity testing is now included as an Organization for Economic Cooperation and Development (OECD) Fish Embryo Toxicity (FET) model [10,11].

In addition to the parameters set out by these guidelines, numerous other toxicity testing protocols and paradigms have been developed that make use of early-stage zebrafish embryos, commonly referred to as Zebrafish Embryo Toxicity (ZET) assays. The majority of these models have been developed as teratogenicity assays that evaluate the effects of potential toxicants on embryo development. A number of different exposure timelines have been used within the first 5 days of development. These include beginning test compound exposure between 4 and 24 h post fertilization (hpf), followed by the assessment of toxicity, often every 24 h, up to 72, 96 or 120 hpf [7,12,13,14,15]. In addition to the teratogenic ZET assays, a toxicity testing model was developed in our lab which exposed larvae to potential toxic compounds beginning at 72 hpf and evaluated visible indicators of toxicity and changes in behaviour at 120 hpf [16]. Since body patterning, organogenesis, and swim bladder inflation is complete by 72 hpf [17], we hypothesize this assay to be more of a general toxicity assay rather than one which evaluates teratogenicity. This assay, termed the General and Behavioral Toxicity (GBT) assay, may then act as a complementary test to the previously developed ZET teratogenic assays.

In the current study we have compared two different embryo/larval exposure paradigms by testing 20 chemicals from different chemical classes/uses and with different mechanisms of action using the GBT assay and a representative teratogenicity ZET assay used previously in our laboratory that exposes larvae from 6–120 hpf. Concentrations that showed a visible phenotype in 50% of the embryos (EC_50_) were compared to the concentrations that showed lethality in 50% of the embryos (LC_50_) for both the ZET and GBT assays. While some compounds showed a visible phenotype and lethality at similar levels in each assay, there were differences, with some compounds showing a higher level of toxicity for the ZET assay and some for the GBT assay. Importantly, when the visible phenotypes produced between the two assays were compared, there were often phenotypic differences, such as the presence of hepatotoxicity, which were only apparent in the GBT assay. Additionally, behavioral testing was performed at 120 hpf for the GBT assay. This testing showed that the lowest observable effect concentration (LOEC) was lower than the visible phenotype-derived LOEC for a subset of the chemicals tested. This would suggest that the behavioral testing may be able to detect underlying effects, such as neurotoxicity, that are not readily apparent through phenotypic screening. This may indicate that behavioral testing is a more sensitive toxicity endpoint than screening phenotypic changes alone.

## 2. Materials and Methods

### 2.1. Animal Husbandry

All zebrafish larvae used were obtained from breeding of wild-type AB/Tub hybrids. Age-matched embryos were sorted for fertilization at roughly 4 hpf and kept in E3 media (5 mM NaCl, 0.17 mM KCl, 0.33 mM CaCl_2_-2H_2_O, 0.33 mM MgSO_4_-7H_2_O) in 10 × 150 mm disposable polystyrene petri dish at 28.5 °C until used in the ZET assay. Embryos to be used in the GBT assay were transferred to Pentair Aquatic Ecosystem (Apopka, FL, USA) nursery baskets (maximum 200 embryos per basket) residing in a 3-L tank in a ZebTec Recirculation Water Treatment System (Tecniplast USA, Easton, PA, USA) and raised until 72 hpf. The water temperature within this system was maintained at 28.5 ± 0.5 °C and the room housing the tank was kept on a 14:10 light: dark cycle. All adult zebrafish husbandry and breeding was in accordance with the Canadian Council of Animal Care (CCAC) guidelines.

### 2.2. Chemicals

The CAS Registry Numbers (CAS RN) and purities of all chemicals tested in the two toxicity assays are listed in Table 1. Testosterone propionate and Dechlorane Plus were purchased from Toronto Research Chemical (Toronto, ON, Canada). All other toxicants and the vehicle, dimethyl sulfoxide (DMSO) (purity ≥ 99%), were purchased from Sigma-Aldrich (Oakville, ON, Canada). Then, 200 mM stocks of the tested chemicals were made in DMSO and stored at −20 °C before use. Dechlorane Plus (12.5 mM) and Testosterone Propionate (180 mM) had lower stock concentrations due to solubility limits. Several other chemicals had stocks with higher concentrations in an attempt to obtain exposure concentrations where 100% lethality could be observed: Amoxicillin (500 mM), Thiabendazole (267 mM) and Resorcinol (527 mM). For both assays described below, working dilutions were made immediately prior to initiating the larval exposures.

### 2.3. Toxicity Assays

#### 2.3.1. Zebrafish Embryonic Toxicity (ZET) Assay

Fertilized zebrafish embryos were transferred to a new polystyrene disposable petri dish and rinsed in HEPES-buffered E3 (HE3) media (5 mM NaCl, 0.17 mM KCl, 0.33 mM CaCl_2_-2H_2_O, 0.33 mM MgSO_4_-7H_2_O, 10 mM HEPES, pH 7.2). Using a 1000 µL wide-bore micropipette tip, embryos were transferred individually into wells of a flat-bottomed 96-well polystyrene tissue-culture treated microtiter plate (Biolite, Fisher Scientific, Toronto, ON, Canada) in a total volume of 270 µL. To initiate the exposure, 30 µL of a 10× stock of chemical in HE3 media was pipetted into the well when the larvae were 6 ± 0.5 hpf. Twelve larvae were exposed per concentration of chemical tested during each replicate along with a carrier control. For all dilutions, the final DMSO concentration was held constant at 0.5% or 1.0% *v/v* (as described in Table 1). Following addition of test chemical, the plates were sealed with ThermoSeal RTS clear-transparent film (Excel Scientific, Victorville, CA, USA) to prevent evaporation and incubated at 28.5 °C on a 14:10 light: dark cycle (light intensity 3–5 µmol m^−2^ s^−1^).

Lethality and phenotypic observations were scored visually on an inverted microscope at 120 hpf ± 0.5 hpf. A larvae was considered dead if there was no observable heartbeat.

#### 2.3.2. General and Behavioral Toxicity (GBT) Assay

Larvae that had been reared in nursery baskets as described above were removed from the recirculation system and rinsed into a disposable 15 × 100 mm polystyrene petri dish in HE3 media. Hatched larvae were placed individually into wells of a 96-well microtiter plate as described for the ZET assay followed by the addition of 30 µL of a 10× stock of test chemical into each well (12 wells per exposure concentration) when the larvae were 72 ± 0.5 hpf. Plates were sealed and incubated as described above.

At 120 hpf, the plates were removed from the incubator and the sealing film removed. Plates were immediately placed in a Noldus Daniovision Behavioral Tracking instrument (Noldus, Leesburg, VA, USA) and observed for 30 min under lighted conditions (15 µmol m^−2^ s^−1^) and a constant temperature of 28.5 °C. Following behavioral tracking, the plates were resealed and visually scored for lethality and affected phenotypes using an inverted microscope.

#### 2.3.3. Dose Curve Fitting/Behavioral Analysis

Exposures for each chemical tested were performed a minimum of three times. Values of % dead and % affected (dead + phenotypically affected) at 120 hpf were plotted against the log of the exposure concentration to determine LC_50_ and EC_50_ respectively using a least squares (ordinary fit) non-linear regression analysis with variable slope (four parameters). All curves were constrained to fit to 0.

Lowest Observable Effect Concentration (LOEC) for the phenotypic endpoints was determined by comparing the % affected (dead + phenotypically affected) values determined for each exposure concentration to that of the vehicle control using a one-way ANOVA followed by a Dunnett’s multiple comparisons test with single pooled variance (*p* < 0.05). The LOEC values for the behavioral observations was statistically determined using the mean of the total distance (mm) travelled under lighted conditions for 30 min for each exposure concentration compared to the vehicle controls. Larvae scored as dead were removed from the behavioral analysis.

## 3. Results

### 3.1. Toxicity Assays

Each of the twenty chemicals listed in Table 1 were screened using both the ZET (6–120 hpf) and GBT (72–120 hpf) assays under similar incubation conditions. Phenotypes (listed in Table 2) were manually scored at 120 h post fertilization (hpf). For the GBT assay, larvae were phenotypically scored immediately following the behavioral testing performed at 120 hpf. Initial exposure concentration range-finding was performed for each chemical using each assay in order to find the concentration at which 100% lethality was observed. The maximal exposure concentrations tested are listed in Table 3. Although it was initially attempted to constrain the DMSO vehicle concentration to 0.5% (*v/v*), for some test chemicals the vehicle concentration had to be increased to 1.0% (*v/v*) in order to achieve 100% affected or 100% lethality. Experimental exposures to the higher DMSO concentration are listed in Table 1.

No significant toxicity was observed for the negative control Amoxicillin for either of the assays at levels up to 2500 and 5000 µM in the ZET and GBT assays respectively. With the exception of Dechlorane Plus, all of the other chemicals tested produced phenotypic responses for both assays including 3,4-Dichloroaniline, which was selected as a positive control. Dechlorane Plus had solubility issues which limited the testable exposure concentrations. This was not surprising as it had the highest Log P values of all twenty chemicals tested in this study.

The initial toxicity profile was generated using a ≥7-point dilution series that was refined to fall between the no observable effect level and that producing 100% lethality or 100% of larvae with a discernable phenotype. The two assays were compared based on the calculated concentration that produced the half maximal effect based on observed phenotypes (EC_50_) and the concentration that produced the half maximal effect based on lethality (LC_50_) (Table 3). A sum of squares F-test was performed to compare the corresponding EC_50_ and LC_50_ values between the two assays.

For all but two of the compounds EC_50_ values obtained from both assays were either statistically the same (as denoted with an asterisk in Table 3) or higher for the GBT assay. A similar trend was observed when comparing LC_50_ values. Most notably, in the case of Bisphenol S, Thiabendazole and Permethrin where although LC_50_ values could be determined in the ZET assay, they could not be determined in the GBT assay. The two exceptions to this overall trend were Benzophenone and Resorcinol, where a greater degree of toxicity was observed in the GBT assay rather than in the ZET assay.

### 3.2. Comparison of Toxicity Ranking Profiles

**Figure 1:**
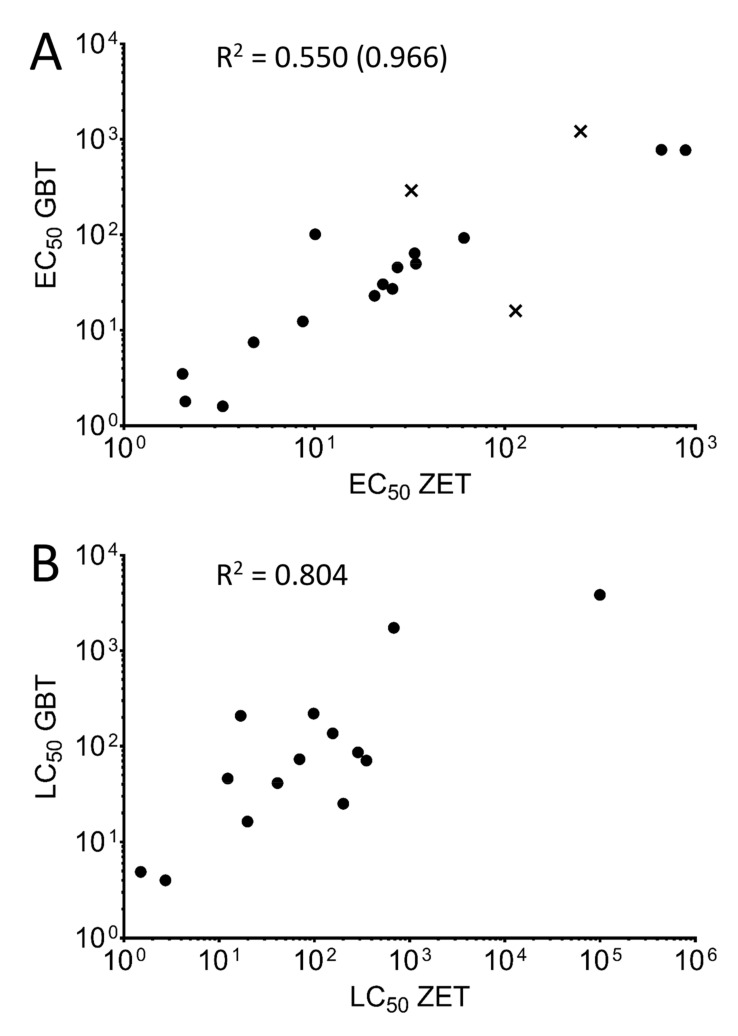
Correlation analysis between assays. The correlation between EC_50_ (**A**) and LC_50_ (**B**) values was determined between the ZET assay (x-axis) and GBT assay (y-axis). Both axes are displayed as log (values) in for ease of visualization. Compounds with no determined toxicity values for one (Benzophenone, Permethrin, and Thiobendazole) or both (Dechlorane Plus, Amoxicillin) of the assays were omitted from the corresponding analyses. Correlation coefficients (R^2^) for both correlation analyses are indicated. Removal of the outlying chemicals (Benzophenone, Valproic acid, and Thiobendazole) from the EC_50_ analysis whose points on the graph are outside the 95% confidence interval of the linear regression (denoted by “×”) results in the correlation coefficient in parentheses.

In order to compare the toxicity rankings of the twenty tested chemicals between the two assays, a correlation analysis was used for both the EC_50_ (Figure 1A) and LC_50_ values (Figure 1B). As Amoxicillin and Dechlorane Plus did not show a measurable level of toxicity for either assay, they were excluded from this analysis.

The toxicity rankings between the chemicals for the EC_50_ and LC_50_ showed a high correlation based on lethality, with a correlation factor of 0.804 when comparing the LC_50_ of fourteen of the eighteen chemicals compared (Figure 1B). A lower correlation was seen when comparing the EC_50_ values for the eighteen chemicals (0.550). This weaker correlation was mostly due to three outlier chemicals falling outside the 95% confidence interval of the linear regression (Benzophenone, Valproic acid, and Thiabendazole, shown in Figure 1A as “×” symbols), as removing these compounds from the analysis increased the correlation coefficient to 0.966.

### 3.3. Comparing Sensitivities of the Two Toxicity Assays

In the current study, the ZET assay incorporates two toxicity endpoints, altered phenotypes and lethality, while the GBT assay incorporates a third measure of toxicity, namely a measure of larval locomotion at 120 hpf. Previous studies have shown that both an increase and a decrease in larval locomotion following prolonged chemical exposure can be linked to potential neurotoxicity [18]. The total distance travelled by the larvae following the 72–120 hpf exposure period was measured for a 30-min period under lighted conditions. The larvae are observed for phenotypic readout following the locomotor measurement and those found to be dead (absence of heartbeat) are excluded from the locomotor activity analysis. The average total distance travelled for the larvae was compared to that of the control larvae for each exposure concentration, and the lowest concentration to produce a statistically significant increase or decrease in activity was considered the lowest observable effect level (LOEC). The statistical values for the behavioral assay are listed in Supplementary Table S1. In the current study 14 of the 18 chemicals that produced a behavioral effect showed a decrease in activity, while exposure to 4 of the chemicals led to an increase in activity as compared to controls. Behavioral LOECs were compared to the phenotypic LOEC values determined from both the ZET and GBT assays (Table 4).

**Figure 2:**
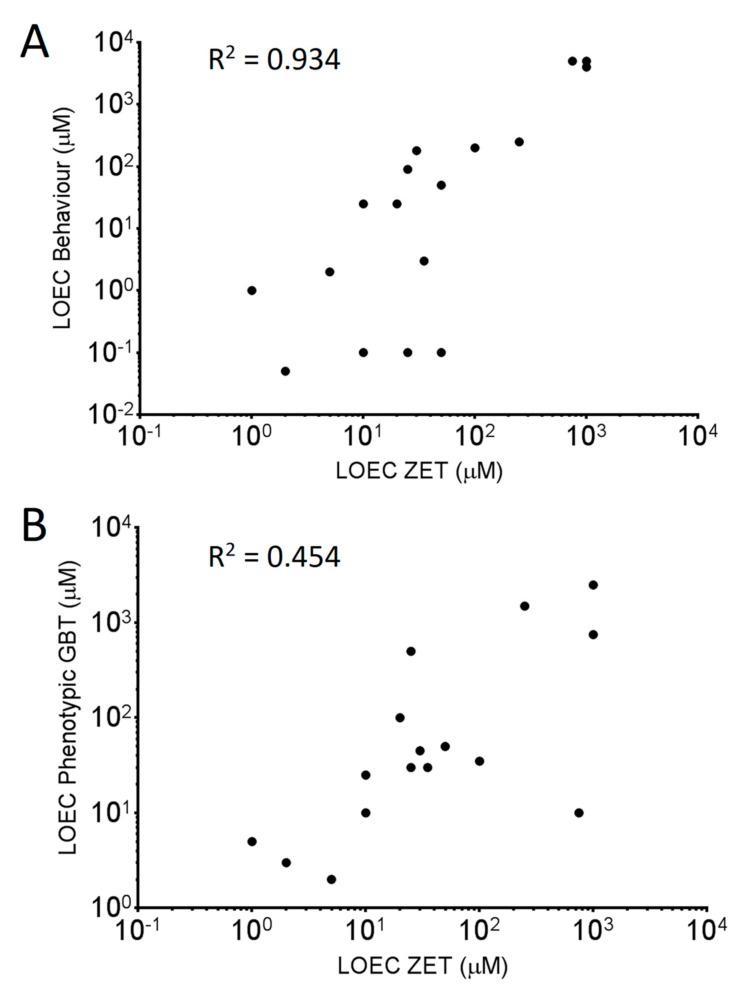
Correlation analysis comparing the LOEC rankings between the ZET assay and the Behavioral endpoint of the GBT assay (**A**) and between the ZET assay and the phenotypic endpoint from the GBT assay (**B**). R^2^ values for each are embedded in the plots.

It was hypothesized that for known neuroactive chemicals, the behavioral LOEC would be lower than the LOEC determined through phenotypic observation. When comparing phenotypic and behavioral LOECs for the GBT assay, ten of the eighteen chemicals with observed toxicity had lower behavioral LOECs than those derived from phenotypic observation. Of those ten, five are known to be neurotoxic (Tricresyl phosphate, Thiabendazole, TBBPA, Aldicarb and Pyriproxyfen) and a sixth (Valproic acid) is a known neuroactive compound (GABA-reuptake inhibitor). Seven of those ten chemicals also exhibited behavioral LOEC values lower or identical to phenotypic LOEC values determined in the ZET assay. Comparing LOEC rankings of the 18 chemicals that presented toxicity (Figure 2) shows that there is a higher correlation between the GBT behavioral LOEC values and the ZET LOEC values (0.934) than between the phenotypic GBT LOEC values and the ZET LOECs values (0.454).

### 3.4. Observed Phenotypes and Phenotypic Profiles

While the EC_50_ values were calculated based on the presence of any visible phenotype, each observed phenotype (as described in Table 2) was also recorded for each living larvae, allowing for more than one phenotype per larvae. Phenotypes were neither weighted based on the exposure concentration in which they were observed nor on the severity of the phenotype. For each chemical, the counted instances of each phenotype within a group (listed in Table 2) were divided by the total number of phenotypic counts observed across all exposure concentrations used to calculate the EC_50_ dose curve for that chemical and phenotype. These percentages were considered a measure of the abundance of each phenotypic group. The phenotypic profiles created for each chemical were used to compare phenotypic abundances for all chemicals within a toxicity assay and also to compare phenotypic profiles of a single chemical between both assays.

**Figure 3:**
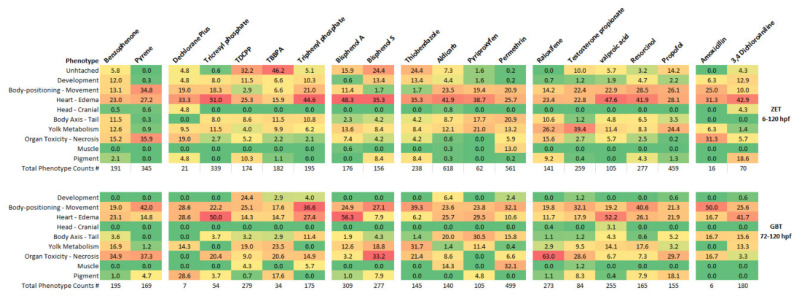
Prevalence of phenotypic groups observed in the ZET (6–120 hpf) and GBT (72–120 hpf) larval toxicity assays for each tested chemical. Phenotypic groups are described in Table 2. The values represent the percentage of times the specified phenotype was observed for all the phenotypic observations of all exposure replicates for each compound. Values are color coded from highest percentage to lowest percentage as red to orange to yellow to green. The total number of phenotypic counts observed for each tested chemical is listed below each column. Note: low phenotypic counts for larvae exposed to Amoxicillin and Dechlorane Plus in both assays.

As shown in Figure 3, the majority of phenotypic observations for all chemicals tested in the ZET assay involved alterations in heart functioning and edema (15.9–50.1%). Altered phenotypes in this group are observed for many larval zebrafish toxicant exposures (reviewed in [19]). The next most prevalent phenotypes in the ZET assay were alterations to body-positioning and movement. In most cases, this involved the observation of a loss in lateral recumbency. Alterations in yolk metabolism were found for a subset of the chemicals tested in the ZET assay, making up more than 10% of the observed phenotypes for Benzophenone, Tricresyl phosphate, Bisphenol A, Aldicarb, Pyriproxyfen, Permethrin, Raloxifene HCl, Testosterone propionate, Valproic acid and Propofol. Alterations in yolk metabolism are well established for endocrine disrupting chemicals such as Bisphenol A [20,21] and for other chemicals disrupting the peroxisome proliferator-activated receptor (PPAR) pathway [22].

As in the ZET assay, the most abundant phenotypes present for the GBT assay belonged to the heart-edema group of phenotypes. The abundance of heart-edema associated phenotypes implies that exposure during the initial body patterning developmental period was not necessary for the occurrence of this group of phenotypes and as such they may be indicators of general toxicity. Effects on cardiac output have been known to create edema in developing larvae [23] so the abundance of edema in larvae exposed at later developmental stages may not be surprising. The second most abundant phenotypes in the GBT assay were that of the body positioning—movement group, as was observed for the ZET assay.

Despite these similarities in phenotypic profiles between the ZET and GBT assays, there were some overall differences. One of the main differences was in the observation of organ toxicity, primarily liver necrosis, which was more prevalent in the GBT assay. While necrosis was seen for some of the chemicals in the ZET assay (Benzophenone, Pyrene, and Raloxifene HCl), several additional chemicals tested with the GBT assay (Tricresyl phosphate, TBBPA, Triphenyl phosphate, Bisphenol S, Thiabendazole, Testosterone propionate, and Propofol) also induced organ toxicity. Organ toxicity was the primary altered phenotype for Bisphenol S and Raloxifene HCl in the GBT assay.

Altered muscle phenotypes were more abundant in the GBT assay than the ZET assay. Following permethrin exposure, muscle abnormalities were present at a 13.0% level in the ZET assay, while its prevalence increased to 32.1% in the GBT assay. Also, while no altered muscle phenotypes were observed following Aldicarb exposures in the ZET assay, these phenotypes made up 13.1% of the total observed phenotypes in the GBT assay.

## 4. Discussion

The zebrafish embryo has become established as a powerful experimental model for testing the teratogenic potential of chemicals and has been proposed as a plausible new approach method (NAM) for testing the toxicity of new chemical entities. The current zebrafish ZET assays used by numerous laboratories often differ in their protocol design. These differences relate to initial exposure time (6–24 hpf), the length of exposure time (48–120 h), chorionated versus dechorionated embryos, and static exposure versus media replacement at multiple time points during the test. A recent study compared some of these different protocols and found that they can and do have an effect on the concentration response pattern of the chemicals tested [24]. However, since the phenotypes produced by the chemicals were similar regardless of the exposure paradigm, the study concluded that the concentration response differences did not affect the conclusions made regarding the bioactivity and potential toxicity of the chemicals tested. In the current study, we tested a Zebrafish Embryonic Toxicity (ZET) exposure paradigm from 6–120 hpf that is similar to those used in some of the previous studies [14,15] and compared it to an exposure period from 72–120 hpf in an assay we term the General and Behavioral Toxicity (GBT) assay [16]. The ZET exposure period was designed as a measure of chemical teratogenicity similar to previous studies, while the GBT assay was designed to act as a general toxicity assay as primary organogenesis is complete and body patterning is largely established by 72 hpf [17]. When the LC_50_ values were compared between the two assays, the majority of the chemicals showed a similar toxicity ranking profile as highlighted by a correlation coefficient (R^2^ values) close to 1. In the case of the EC_50_ toxicity ranking comparisons, differences were found between the ZET and GBT values for Benzophenone, Valproic acid and Thiabendazole. Removing these chemicals from the R^2^ calculation moved the value from 0.55 to 0.96, suggesting the EC_50_ profiles for the other chemicals tested were similar. For these three outlier chemicals, the ZET assay showed a higher sensitivity to Valproic acid and Thiabendazole, while the GBT assay was more sensitive to Benzophenone. Interestingly, although the phenotypes produced by exposure to Valproic acid were similar between the ZET and GBT assays, there were notable differences for Benzophenone and Thiabendazole. Organ necrosis (primarily the liver) and yolk metabolism represented a greater proportion of the affected phenotypes in the GBT assay compared to the ZET assay for both Benzophenone and Thiabendazole. Taken together, these findings suggest that the differences in sensitivity between the two assays were chemical specific.

In addition to Benzophenone and Thiabendazole, there were other chemicals whose phenotypic profiles differed between the ZET and GBT assays. For example, although liver necrosis was observed in the ZET assay for Benzophenone, Pyrene and Raloxifene HCl, a higher level of liver necrosis was observed in the GBT assays for Bisphenol S, Raloxifene HCl, TBBPA, Propofol, Testosterone Propionate, Tricresyl phosphate and Triphenyl phosphate. This suggests that the GBT assay may improve the detection of potential hepatotoxicity.

It has been previously shown that changes in larval behaviour can be used as an indicator of neurotoxicity [7,8,25]. These studies tested larval behaviour following similar exposure paradigms to the aforementioned ZET assays. The behavioral testing conducted in this study was done following the GBT exposure paradigm from 72–120 hpf using a simplified measure of baseline activity during 30 min in a lit environment. We compared the lowest observable effect concentration (LOEC) as determined by behavioral testing to that of the ZET and GBT phenotype assays. We found that for 7 of the 20 compounds tested, the LOEC for the behavioral assay was lower than for both the ZET and GBT phenotype assays. Of those chemicals, five (Tricresyl phosphate, Thiabendazole, TBBPA, Aldicarb and Pyriproxyfen) have been found to display neuroactive/neurotoxic side effect profiles in other models [4,26,27], with Tricresyl phosphate and Pyriproxyfen linked to polyneuropathy and microcephaly in humans [28,29]. Thus, the GBT behavioral testing paradigm appears to highlight chemicals that have neurotoxic potential and was able to provide a more sensitive toxicity measurement than phenotypic observation alone. Using the LOEC values it was also able to create a toxicity ranking profile closer to that of the ZET assay (R^2^ = 0.934) than the GBT phenotypic assay (R^2^ = 0.454). The correlation of GBT behavioral LOEC values and ZET phenotypic LOEC values of all chemicals tested was better than the comparison of EC_50_ values when outliers were removed.

Previous studies that have made use of locomotor changes in response to stimuli such as the “dark startle” [7,26] and vibration response [5,30,31] have highlighted the ability of changes in larval behaviour to act as indicators of neurotoxicity. Further behavioral work incorporating these types of stimuli following the exposure period for the GBT assay may further increase the sensitivity of the behavioral assay and lead to better identification of neuroactive toxicants. These assays will be incorporated into future studies using the GBT model.

While there are overall similarities in the toxicity profiles between the ZET and GBT assays, there are notable differences that are most certainly a reflection of the physiologies of the life stages covered by each assay. The ZET assay is primarily a measure of organogenesis, while the GBT assay the impact of chemicals on later stages of development and on locomotor behaviour. Since the GBT assay can detect similar toxicity profiles to that of the ZET assay, while potentially providing additional information on targets of toxicity that may not be discernable with the ZET assay, this model will complement ZET testing and help to strengthen the use of the zebrafish embryo as a new approach method for chemical testing.

## Figures and Tables

**Table 1:** Groupings of chemicals tested in this study. Chemicals are grouped by use and span a wide range of molecular weights and Log *p* values. All compounds were tested in the presence of dimethyl sulfoxide (DMSO) as a carrier with the percentage of DMSO used for each compound listed.

Type	Compound Name	CAS RN	Purity	MW (g/mol)	Use	Log *p*	DMSO Vehicle % (*v/v*)
Chemical precursors	Pyrene	129-00-0	99%	202.25	precursor for dyes, plastics, and pesticides	4.9	1.0%
	Benzophenone	119-61-9	≥99%	182.22	UV blocker, flavour ingredient, fragrance enhancer	3.4	0.5% (ZET)/1.0% (GBT)
Flame retardants	Dechlorane Plus	13560-89-9	98%	653.7	flame retardant	8	1.0%
	Tricresyl phosphate (TCrP)	1330-78-5	90%	368.4	flame retardant	5.1	1.0%
	Tris(dichloro-isopropyl) phosphate (TDCPP)	13674-87-8	≥95%	430.9	flame retardant	3.3	0.5%
	3,3′,5,5′-tetrabromobisphenol A (TBBPA)	79-94-7	97%	543.9	brominated flame retardant	6.8	0.5%
	Triphenyl phosphate (TPhP)	115-86-6	≥99%	326.28	plasticizer and flame retardant	4.6	0.5%
Plasticizers	Bisphenol A	80-05-7	≥99%	228.29	plasticizer	3.3	0.5%
	Bisphenol S	80-09-1	>98%	250.27	plasticizer	1.9	1.0%
Pesticides/Fungicides	Thiabendazole	148-79-8	>99%	201.25	veterinary fungicide	2.5	0.5%
	Aldicarb	116-06-3	≥98%	190.27	pesticide	1.1	1.0% (ZET)/0.5% (GBT)
	Pyriproxyfen	95737-68-1	≥98%	321.4	veterinary drug for flea control	4.8	1.0%
	Permethrin	52645-53-1	≥90%	391.3	human and veterinary insecticide (head lice and scabies)	6.5	0.5%
Pharmaceuticals	Raloxifene HCl	82640-04-8	>99%	510	treatment of osteoporosis and breast cancer	6.1 (non-ionic version)	0.5%
	Testosterone propionate	57-85-2	98%	344.5	anabolic steroid, treatment of breast cancer	4.4	0.5% (ZET)/1.0% (GBT)
	Valproic acid	99-66-1	>97.5%	144.21	anticonvulsant	2.8	0.5%
	Resorcinol	108-46-3	≥99%	110.11	topical treatment of skin disorders	0.8	1.0%
	Propofol	2078-54-8	97%	178.27	sedative	3.8	0.5%
Controls	Amoxicillin	26787-78-0	potency: ≥900 μg per mg	365.4	antibiotic, negative control	−2	0.5% (ZET)/1.0% (GBT)
	3,4-Dichloroaniline	95-76-1	98%	162.01	positive control in OECD FET	2.7	0.5%

**Table 2:** Phenotypes and phenotypic groupings used for manual scoring of larvae at 120 h post fertilization (hpf) for both the Zebrafish Embryo Toxicity (ZET) and General and Behavioral Toxicity (GBT) assays.

Phenotypic Group	Phenotypes
Development	Gross morphological abnormality
	Overall short body
Body positioning—Movement	Loss of lateral recumbency
	Constant movement
Heart—Edema	Slow heart rate
	Pericardial edema
	Blood pooling
	Yolk syncitial layer edema
	Head edema
	Large gut lumen
Head—Cranial	Small head
	Gray/cloudy head
Body axis—tail	Curved tail—dorsal/ventral axis
	Curved tail
Yolk metabolism	Large yolk (for developmental time point)
	Gray/cloudy yolk
Organ toxicity—necrotic	Dark coloured liver
	Dark coloured nose/mandible
Muscle	Ruffled tail fin
Pigment	Lighter pigment
	Overall darker colour
	Melanocyte aggregation

**Table 3:** Summary of EC_50_ and LC_50_ data for both the ZET (6–120 hpf exposure) and GBT assays (72–120 hpf exposure). For each compound the EC_50_ and LC_50_ dose curves from the two assays were statistically compared using an extra sum of squares F-test (*p* > 0.05). Curves from both the ZET and GBT assays that are statistically similar are denoted with an asterisk (*). All values are shown in µM and a hyphen (-) denotes that an EC/LC_50_ value could not be determined. The maximum exposure concentration tested in both assays is listed.

	EC_50_ (µM)		LC_50_ (µM)		Maximum Exposure Concentration (µM) Tested	
	ZET	GBT	ZET	GBT		
	6–120 hpf	72–120 hpf	6–120 hpf	72–120 hpf	ZET	GBT
Benzophenone	114	16	-	787.9	5000	5000
Pyrene	33.6 *	64.1 *	156 *	136.8 *	2000	2000
Dechlorane Plus	-	-	-	-	62.5	62.5
Tricresyl Phosphate	20.7 *	23 *	201.2 *	25.1 *	2000	500
TBBPA	2.03	3.5	2.7	4.0	50	10
TDCPP	8.7 *	12.4 *	12.3	45.9	500	500
Triphenyl phosphate	4.8 *	7.5 *	19.8	16.4	50	50
Bisphenol A	34.1 *	49.8 *	69.8 *	73.2 *	100	250
Bisphenol S	664 *	778 *	1722	-	4000	2500
Thiobendazole	32.3	291	59.0	-	90	1000
Aldicarb	2.1	1.8	1.5	4.9	2000	500
Pyriproxyfen	60.99	92.8	98.2	220.1	500	500
Permethrin	3.3	1.6	-	-	50	10
Raloxifene HCl	25.7 *	27.2 *	41.0 *	41.2 *	90	75
Testosterone Propionate	27.3	45.7	286.7	86.4	900	450
Valproic Acid	250	1215	682.2	1743	2500	2500
Resorcinol	888.4 *	771 *	-	3849	5270	5000
Propofol	22.9 *	30.4*	351.6	70.9	1000	500
Amoxicillin	-	-	-	-	2500	5000
3,4-Dichloroaniline	10.1	101.4	16.8	208.7	500	500

**Table 4:** Lowest observed effect concentrations (LOEC) for each compound based on the phenotypic observations in the ZET/GBT assays and any change in behaviour for the GBT assay. All values are expressed in µM. LOEC values for the behavioral changes of the GBT assay marked with an asterisk (*) denote LOEC concentrations where the distance travelled by exposed larvae was higher than the carrier controls. The statistical values for the behavioral LOEC calculations are included as Supplementary Data (see Supplementary Table S1).

	Lowest Observed Effect Concentration (LOEC) (µM)		
	ZET	GBT	GBT
	(6–120 hpf)	(72–120 hpf)	Distance Travelled
Benzophenone	750	10	5000
Pyrene	50	50	50
Dechlorane Plus	-	-	-
Tricresyl Phosphate	25	30	0.1 *
TDCPP	10	25	25
TBBPA	2	3	0.05
Triphenyl phosphate	10	10	0.1 *
Bisphenol A	50	50	50
Bisphenol S	1000	750	5000
Thiobendazole	25	500	90
Aldicarb	1	5	1
Pyriproxyfen	50	50	0.1
Permethrin	5	2	2 *
Raloxifene HCl	35	30	3 *
Testosterone Propionate	30	45	180
Valproic Acid	250	1500	250 *
Resorcinol	1000	2500	4000
Propofol	100	35	200
Amoxicillin	-	-	-
3,4-Dichloroaniline	20	100	25

## Supplementary Materials

The following are available online at https://www.mdpi.com/2305-6304/8/4/126/s1, Table S1: Calculated behavioral lowest effected concentration (LOEC) values.
