# Supplementary material for: Comparison of the Zebrafish Embryo Toxicity Assay and the General and Behavioral Embryo Toxicity Assay as New Approach Methods for Chemical Screening
Source: Toxics. 2020 Dec 21;8(4):126. doi: 10.3390/toxics8040126 (PMC7767334; doi:10.3390/toxics8040126)
Supplement: Supplementary file 1 [file toxics-08-00126-s001.pdf]

toxics

Supplementary Material: Comparison of the Zebrafish Embryo Toxicity Assay and the General and Behavioral Embryo Toxicity Assay as New Approach Methods for Chemical Screening

John C. Achenbach [1], Cindy Leggiadro [1], Sandra A. Sperker [1], Cindy Woodland [2] and Lee D. Ellis [2,*]

Compound	LOEC (µM)	total distance travelled (mm)	S.E.M.	N	Significance
Permethrin	2	**4942**	568.9	71	*
Bisphenol S	5000	0	0	48	
Benzophenone	5000	700.9	271	13	**
Raloxifene HCl	3	**2500**	206.7	36	****
Thiobendazole	90	1348	96.86	35	*
TDCPP	25	448.5	95.83	40	****
3,4-Dichloroaniline	25	490.2	159.6	21	***
TBBPA	0.05	1317.0	155.4	34	**
Triphenyl phosphate	0.1	**2235**	184.9	42	*
Resorcinol	4000	657	200.9	19	*
Testosterone propionate	180	0	0	36	
Amoxicillin	-	-	-	-	
Valproic acid	250	**2389**	168.4	35	**
Propofol	200	0	0	36	
Bisphenol A	50	1383	158.1	41	*
Tricresyl phosphate	0.1	**1751**	217	36	*
Dechlorane Plus	-	-	-	-	
Pyriproxifen	0.1	1171	141.6	36	**
Pyrene	50	522.5	117.9	36	***
Aldicarb	1	790.4	111.8	36	**

Table S1. Calculated behavioural lowest effected concentration (LOEC) values. The mean total distance travelled in 30 min under lighted conditions for living larvae at each exposure concentration was compared to vehicle controls using a one-way ANOVA followed by a Dunnett's multiple comparisons test with single pooled variance. For each LOEC the standard error of the mean (S.E.M.) value is indicated along with the number of embryos (n) and the statistical significance from vehicle control (* p <0.0332, ** p < 0.0021, *** p < 0.0002, **** p < 0.0001). Distance travelled values larger than their corresponding vehicle controls are indicated in boldface. Hyphens (-) denote that a LOEC could not be calculated as there was no statistical significance observed between any exposure concentration and the vehicle control.
